# Hepatocellular Carcinoma in a Patient With Crohn’s Disease

**DOI:** 10.7759/cureus.16570

**Published:** 2021-07-22

**Authors:** Akira Hokama, Shingo Arakaki, Shinichiro Ishino, Yutaka Nakagawa, Souhei Tabata, Daiki Agarie, Satoshi Kuwae, Ryuta Zukeyama, Tatsuji Maeshiro, Yuma Tsuruta, Akiko Matsuzaki, Naoki Wada, Mitsuhisa Takatsuki, Jiro Fujita

**Affiliations:** 1 Department of Endoscopy, Graduate School of Medicine, University of the Ryukyus, Nishihara, JPN; 2 Department of Infectious, Respiratory, and Digestive Medicine, Graduate School of Medicine, University of the Ryukyus, Nishihara, JPN; 3 Department of General and Digestive Surgery, Graduate School of Medicine, University of the Ryukyus, Nishihara, JPN; 4 Department of Pathology, University of the Ryukyus Hospital, Nishihara, JPN; 5 Department of Pathology and Oncology, Graduate School of Medicine, University of the Ryukyus, Nishihara, JPN

**Keywords:** hepatocellular carcinoma, infliximab, pathology, computed tomography, crohn’s disease (cd)

## Abstract

A 44-year-old woman with a 26-year history of Crohn’s disease (CD) presented with intermittent fever, vomiting, and watery diarrhea. Her medication included an elemental diet, mesalazine, and infliximab. Liver profile and viral hepatitis markers were normal. Computed tomography scans showed a hepatic tumor by chance. Serum tumor markers disclosed elevated protein induced by vitamin K absence-II. With a diagnosis of hepatocellular carcinoma (HCC), she underwent a hepatic resection of the tumor, revealing well-to-moderately differentiated HCC. The nontumor region of the liver disclosed the absence of cirrhosis or other diseases. Here, the development of HCC in CD without underlying liver diseases is discussed with a review of the literature.

## Introduction

The development of hepatocellular carcinoma (HCC) is mostly associated with underlying liver diseases, such as cirrhosis and chronic hepatitis. Crohn’s disease (CD) is often complicated with various hepatobiliary disorders, including cholelithiasis and abnormal liver function [1]. Recently, several cases of HCC have been reported among CD patients without cirrhosis, and immunosuppressive treatments for CD have been focused on as potential carcinogenic factors. Here, we present the case of a patient with CD who developed HCC in the absence of underlying liver diseases and discuss the background of carcinogenesis.

## Case presentation

**Figure 1:**
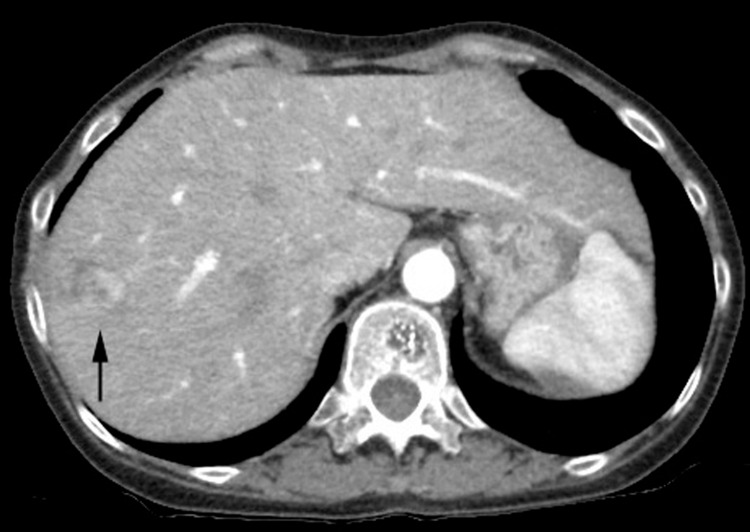
Abdominal CT. CT scan showing a heterogeneously enhancing hepatic tumor (arrow) at the arterial phase. CT: computed tomography

A 44-year-old woman with a 26-year history of CD presented with intermittent fever, vomiting, and watery diarrhea. Her medical history was significant for a colonic resection at the age of 40 and a proctocolectomy with end ileostomy at the age of 41. Her medication included an elemental diet, mesalazine 3 g daily, and infliximab (IFX) 10 mg/kg every eight weeks. She denied smoking and alcohol use. Physical examination revealed stable vital signs and a body mass index of 17.9 kg/m^2^. The abdomen was soft and nontender. Laboratory tests showed white blood cells of 5,700/μL, hemoglobin of 10.1 g/dL, platelets of 32.5 × 10^4^/μL, and C-reactive protein of 0.85 mg/dL (reference range: <0.23 mg/dL). Liver profile and viral hepatitis markers were normal: bilirubin 0.4 mg/dL (reference range: 0.4-1.5 mg/dL), aspartate aminotransferase 20 U/L (reference range: 13-30 U/L), alanine aminotransferase 35 U/L (reference range: 7-23 U/L), alkaline phosphatase 158 U/L (reference range: 106-322 U/L), lactate dehydrogenase 120 U/L (reference range: 124-222 U/L), and gamma-glutamyl transferase 11 U/L (reference range: 9-32 U/L). Computed tomography (CT) scans were performed to evaluate the symptoms, which showed a hepatic tumor incidentally. A CT scan showed a heterogeneously enhancing hepatic tumor at the arterial phase (Figure 1).

**Figure 2:**
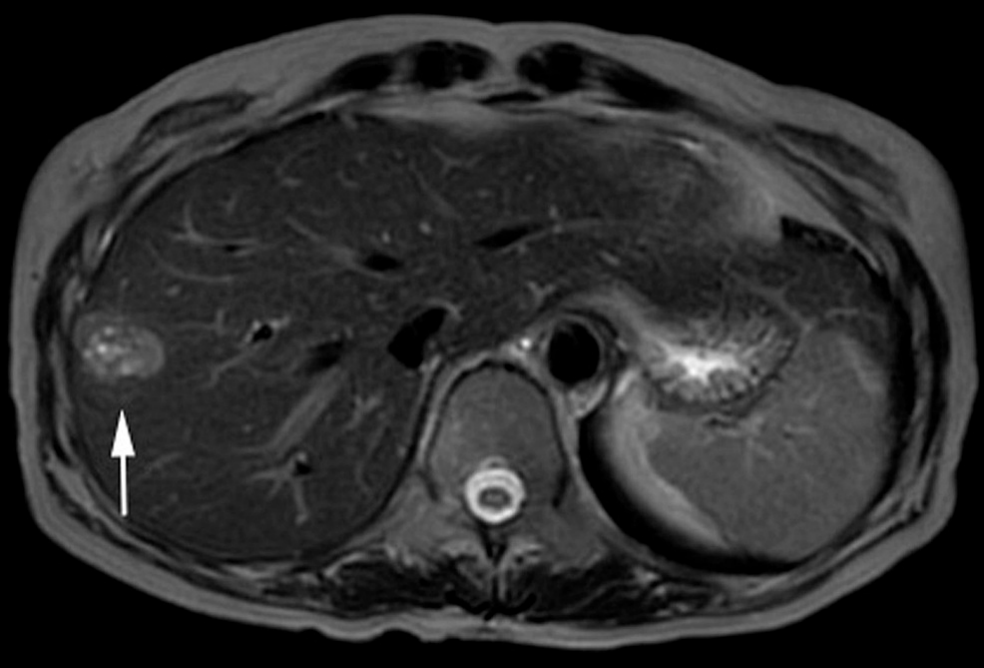
Abdominal MRI. Gadolinium ethoxybenzyl diethylenetriamine pentaacetic acid-enhanced MRI showed the 28-mm-sized hepatic tumor (arrow) on T2-weighted imaging. MRI: magnetic resonance imaging

Gadolinium ethoxybenzyl diethylenetriamine pentaacetic acid-enhanced magnetic resonance imaging showed the hyperintense tumor on T2-weighted imaging (Figure 2).

**Figure 3:**
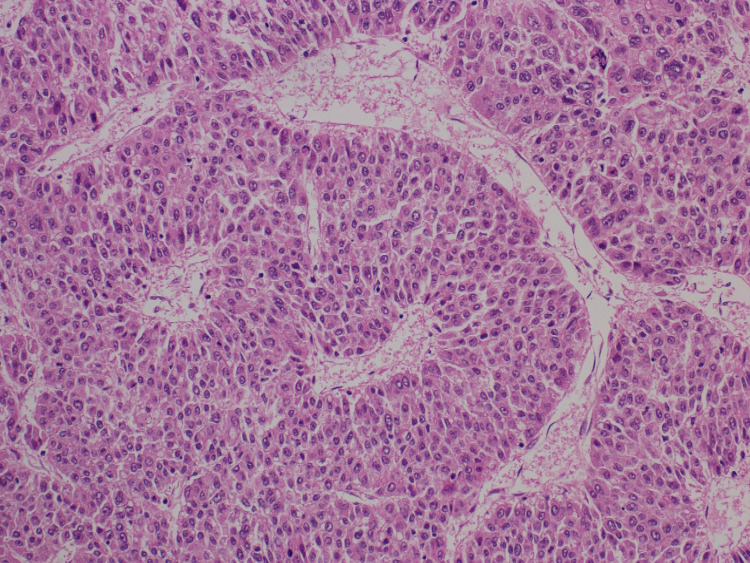
Histopathological figure. Histopathological findings of the tumor showed well-to-moderately differentiated HCC without evidence of microvascular invasion (hematoxylin and eosin staining, magnification: ×200). HCC: hepatocellular carcinoma

**Figure 4:**
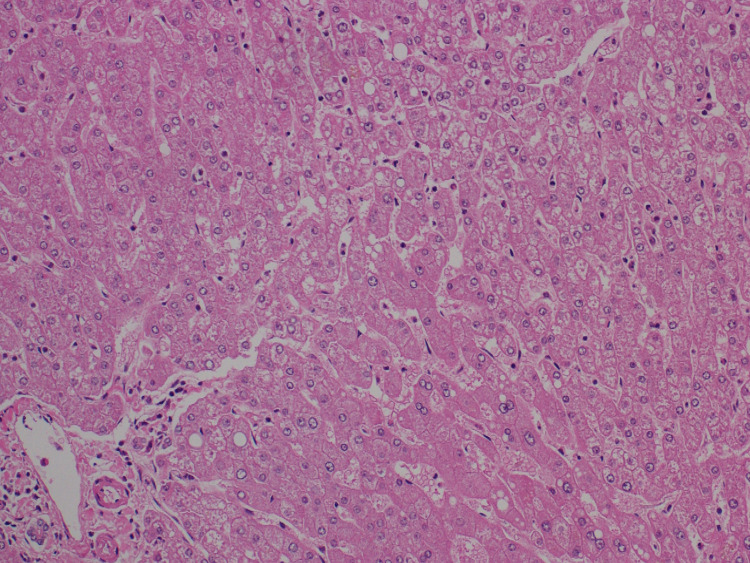
Histopathological figure. The nontumor region of the liver disclosed the absence of cirrhosis or other diseases (magnification: ×200).

Serum tumor markers showed α-fetoprotein of 7 ng/mL (reference range: <9 ng/mL) and elevated protein induced by vitamin K absence-II of 1,411 mAU/mL (reference range: <40 mAU/mL). HCC was suspected based on laboratory and imaging studies. She underwent a hepatic resection of the tumor with an uncomplicated postoperative course. Pathological examination of the tumor showed well-to-moderately differentiated HCC without evidence of microvascular invasion (Figure 3). The non-neoplastic liver showed no evidence of chronic hepatitis, liver cirrhosis, or other metabolic disorders (Figure 4).

IFX was discontinued because of the potential effects in post-cancer surgery and vedolizumab was initiated. The patient has been free from recurrence for two years.

## Discussion

Most HCC cases develop in the presence of cirrhosis or hepatitis. This case presents important clinical issues that HCC can occur in patients with CD, even without underlying liver diseases for HCC. The occurrence of HCC among patients with CD is extremely rare, with only 15 cases, including our case, published as case reports in the literature [2-15].

Table 1 summarizes the clinicopathological features of HCC in CD patients. The average age of the diagnosis of HCC among these CD patients was 40.6 years, which was younger than that of usual HCC (50 years) [16]. The average period between the onset of CD and the diagnosis of HCC was 19.3 years. Several hepatic diseases have been reported in association with CD, which include cholelithiasis, primary sclerosing cholangitis (PSC), steatosis, and drug-induced abnormal liver function [1]. A total of eight (53.3%) cases, including the present case, had no underlying liver diseases; whereas two cases had PSC, four cases had focal hepatic glycogenosis (FHG), and one case had chronic liver inflammation. It is well known that both PSC and FHG have significant neoplastic potentials.

**Table 1:** Clinicopathological features of HCC in patients with CD. CD: Crohn’s disease; AFP: alfa-fetoprotein; PIVKA-II: protein induced by vitamin K absence-II; HCC: hepatocellular carcinoma; AZA: azathioprine; PSL: prednisolone; NA: not available; DOD: died of disease; 5-ASA: 5-aminosalicylic acid; FHG: focal hepatocyte glycogenosis; LT: liver transplantation; PSC: primary sclerosing cholangitis; TACE: transcatheter arterial chemoembolization; ADA: adalimumab

Case No.	Gender	Age: onset of CD/discovery of HCC	Therapy for CD	Serum AFP/PIVKA II (ng/mL, mAU/mL)	Pathology of HCC	Pathology of non-neoplastic liver	Therapy and outcome	Reference No.
1	F	29/43	AZA, PSL	NA/NA	NA	No cirrhosis	DOD with carcinomatosis	[2]
2	F	9/22	5-ASA, AZA	55,000/NA	Trabecular	No cirrhosis positive FHG	Recurrence 6 months after surgery	[3]
3	M	13/33	5-ASA, AZA	NA/NA	NA	No cirrhosis	Lung metastasis	[4]
4	F	63/63	5-ASA	NA/1,100	NA	PSC	No recurrence 15 months after LT	[5]
5	F	14/28	AZA, IFX	26.9/NA	Trabecular, pleomorphic	No cirrhosis positive FHG	Surgery	[6]
6	M	17/33	AZA	Normal range/NA	NA	PSC	DOD with carcinomatosis	[7]
7	M	19/37	5-ASA, AZA, PSL	15/NA	Trabecular to sinusoidal, pleomorphic	NA	DOD 3 months after onset	[8]
8	M	16/52	5-ASA	13.9/16,300	Trabecular	Chronic liver inflammation	Recurrence after TACE	[9]
9	M	13/25	AZA, IFX, PSL	78/NA	Pleomorphic	No cirrhosis positive FHG	No recurrence 1 year after surgery	[10]
10	M	29/37	5-ASA, AZA, PSL	7.7/757	Pseudoglandular	No cirrhosis positive FHG	No recurrence 2 years after surgery	[11]
11	M	9/34	AZA, IFX	3307/NA	Trabecular	No cirrhosis	Carcinomatosis; died 5 months after	[12]
12	M	20/58	5-ASA, AZA	10.3/NA	Moderately differentiated	No cirrhosis	Recurrence 4 years after surgery	[13]
13	M	20/40	IFX, ADA	NA/NA	Moderately differentiated	No cirrhosis	Recurrence 5 years after surgery	[14]
14	F	31/61	5-ASA, AZA, IFX	NA/NA	Well-differentiated	No fibrosis	No recurrence 11 months after surgery	[15]
15	F	18/44	5-ASA, IFX	7/1,411	Moderately differentiated	No cirrhosis	No recurrence 2 years after surgery	Present case

Considering the association with the treatments for CD, 11 (73.3%) cases were treated with AZA, and six (40%) cases were administered IFX. Although AZA has an increased risk of neoplasms including lymphoma and skin cancer, the association with HCC is unknown. The effects of IFX on the development of HCC have not been clarified. Several epidemiology investigations revealed no association between IFX and cancer risks [17,18]. Therefore, IFX may be an innocent bystander in HCC among patients with CD [12]. Although acute liver injury caused by immunosuppressants and biologics has been well studied in the treatment for CD [19], long-term hepatotoxicity and hepatocarcinogenesis should be further monitored carefully. In addition, a recent Swedish/Danish population-based cohort study (1969-2017) revealed 28 deaths from HCC among 47,399 patients with CD and corresponding hazard ratios (HRs) of 1.96 (95% confidence interval: 1.31-2.93) [20]. Although this data lacked information on smoking, alcohol consumption, and underlying hepatic diseases, specific surveillance strategies for HCC was needed because of high HRs.

## Conclusions

In summary, we present a case of HCC in a patient with CD who was treated with IFX in the absence of underlying liver diseases. Although the precise pathophysiology of the development of HCC in CD has not been clarified and potential links should be further investigated, the liver should be surveyed closely by imaging studies in addition to routine blood tests in the era of immunosuppressive treatments with AZA or biologics.
